# Conceptualizing the social networks of children of parents with serious mental illness: a thematic analysis

**DOI:** 10.3389/fpsyg.2024.1383532

**Published:** 2024-07-23

**Authors:** Imogen Nevard, Judith Gellatly, Helen Brooks, Penny Bee

**Affiliations:** Division of Nursing, Midwifery and Social Work, Faculty of Biology, Medicine and Health, School of Health Sciences, The University of Manchester, Manchester, United Kingdom

**Keywords:** children of parents with severe and enduring mental illness (COPMI), parental mental health, social network analysis (SNA), qualitative social network analysis, child network

## Abstract

**Aims:**

Social networks, defined as the set of active and significant ties surrounding an individual, influence the wellbeing of vulnerable children. The best evidenced mechanism through which this occurs is where networks act as a vehicle to access social support. Little is known about the content and function of social networks of children of parents with severe and enduring mental illness (COPMI). COPMI are a frequently under-identified vulnerable child population at risk of negative outcomes. This qualitative study investigates the structure, role and function of these children’s networks.

**Methods:**

Researchers conducted 17 semi-structured egocentric social network interviews. Interviews incorporated personal network mapping as a data collection method. COPMI were recruited through third sector organizations and interviewed across three sites in England. Data was analyzed using an inductive thematic analysis.

**Results:**

Five network features were identified (i) parents as primary providers of support (i) limited networks and diminished connections over time (iii) substitutable ties (formal and informal) (iv) peer connections as source of both support and strain (v) coping strategies: self-censorship, avoidance and animals.

**Conclusion:**

Children of parents with severe and enduring mental illness networks are structurally typical of vulnerable children in that they are limited, rely on parents as primary ties but allow for some substitution of support ties. COPMI-specific features included peer relationships at times as source of strain and network level coping strategies used to manage wellbeing, including pets. This latter reflects previous findings in vulnerable adult populations so far unevidenced in children. Little evidence as to the mechanistic effect at work within networks was collected. However, COPMI were clearly shown to be engaged in active management and strategising in network navigation approaches, indicating the need to engage with children in this capacity, rather than approaching them as passive recipients of support. As such, effective network level interventions for this group are likely to prioritize access to beneficial substitute ties when support is limited. Additionally, interventions that promote network navigation skills and help foster productive coping strategies can capitalize on the child’s active management role within their network.

## 1 Introduction

Children of parents with severe and enduring mental illness (COPMI) are a vulnerable child group at risk of adverse outcomes including psychiatric disorder, poor physical health, welfare concerns and negative behavioral, educational and psychosocial outcomes including social isolation (Oyserman et al., 2000; Foster et al., 2005; Nicholson et al., 2008; van Santvoort et al., 2015; Dreyer et al., 2018; Breslend et al., 2019). International systematic reviews find that over half of adults with severe and enduring mental illness (SMI) are parents, and around a third of adults accessing inpatient or community mental health care are parents (Reupert and Maybery, 2016).

A recent systematic review demonstrates the positive influence of social networks upon the wellbeing of vulnerable children (Nevard et al., 2021). The review identified a lack of qualitative social network data available for COPMI in particular however. In general, social network analysis is an underutilized method in child populations (Salzinger et al., 2015). This study presents a qualitative social network analysis of this demographic and aims to investigate the ways in which COPMI networks cohere and differ from those of other vulnerable child groups. Vulnerable children overall have impoverished social networks in terms of structure and function. The exception to this is in the case of vulnerabilities related to ethnic minority status, where networks were not found to be of a lesser quality. Network embeddedness, defined as the presence of a structure of close social ties able to facilitate resource flow around an individual, is associated with positive outcomes. The association between network embeddedness and positive outcomes is particularly strong for homeless child populations (Nevard et al., 2021). Families are the primary providers of support where available. Large, diverse networks allow for a substitutability of ties if parental support is not available or sufficient i.e., access to supportive alternate ties can compensate for limitations in parenting. It can be hypothesized that some of these features will hold true for COPMI, especially those related to limited parental capacity. However some unique network features to this population that differ from vulnerable children in aggregate, may exist due to the specific nature of being a child of a parent experiencing SMI.

This qualitative study uses egocentric social networks (ego-net) as a conceptual framework for examining the social context of individuals. This means that networks are observed from the point of view of a specific individual at the nucleus of a wider social network, rather than mapping all network ties in a community. Therefore, only individuals with a direct tie to the individual of interest will be recorded on the network map. A social network is defined for the purposes of this study as a personal community: the set of active and significant ties which are important to an individual’s everyday life (Vassilev et al., 2014). Ego-net analyses collate data on the structure, function, and composition of network ties of multiple individuals from the population of interest. Qualitative analyses such as the one utilized in this study use interview data alongside personal network mapping methods to describe how social networks contribute to an individual’s quality of life and wellbeing. This provides information on the quality as well as quantity of network ties. It also allows interviewees, in this case COPMI, to describe their perceptions of network effects i.e., the impact of behaviors within the network by network members. This allows researchers to better conceptualize the role and impact of network ties on wellbeing and quality of life of these children.

This study aims to provide a qualitative description of the characteristics, role and function of social networks for COPMI based on an inductive thematic analysis of themes designed to generate network features. Specific objectives include (i) to collect network data using personal mapping and network interview techniques from COPMI (ii) to thematically analyze this empirical data to generate insights as to the role, structure, function, quality and characteristics of networks for COPMI. The study did not set out to prove or disprove any specific hypotheses about COPMI networks, but anticipated encountering notions such as social support, social stigma, friendship, trust and network related coping strategies based on findings from prior literature. A specific methodological objective was (iii) to collect data directly from children to prioritize the phenomenological perspective of the child rather than relying on adult proxies. This was an ethically informed choice to amplify children’s voices and situate them as experts on their own lives with valuable contributions to research.

## 2 Materials and methods

### 2.1 Recruitment

The sample was recruited via advertisement in third sector organizations working with vulnerable children including COPMI as service users. These included community and young carer groups as well as family interventions. Participants responded directly to a physical or digital advertisement distributed by the organization on their premises or via email or social media, or were approached directly by the organization.

Inclusion criteria for participation were that the child was aged between 6 and 17 years and spent 10 h minimum per week with a parent experiencing SMI. For the purposes of this study SMI was defined as a psychiatric diagnosis of chronic symptomatology (minimum 1 year duration) with significant impact on daily functioning. Commonly reported diagnoses included bipolar disorder, personality disorders and post-traumatic stress disorder. Inclusion criteria were adapted to include self-report from parents. This was because intermediaries in organizations advised that access to secondary mental health services was not a reliable indicator of severity of illness as parents commonly reported difficulties accessing appropriate mental health care despite severe symptomatology.

Consent forms were completed by parents after reviewing relevant participant information materials if children were under 16 years of age. Young people aged 16 or 17 gave consent on their own behalf. Children under 16 completed an assent form immediately prior to interview, detailing the central features of participation in order to promote understanding of the process. This was again an ethical consideration related to prioritizing children in the research process rather than deferring to parental choice. No families, children or young people refused consent or exited the study early.

### 2.2 Data collection

**FIGURE 1:**
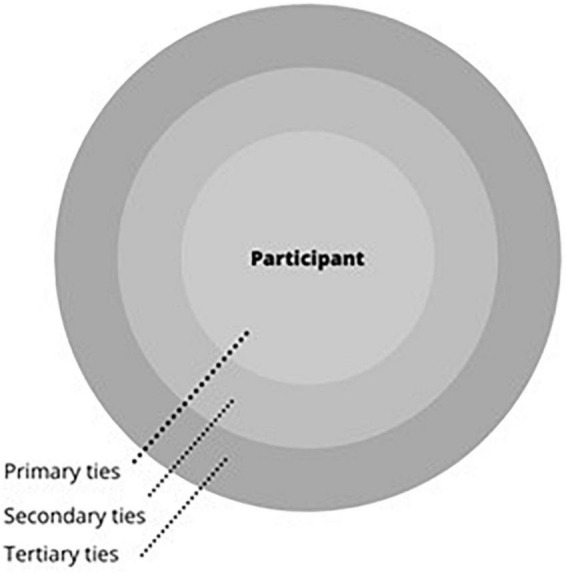
Template network diagram.

Thirty-minute, semi-structured ego-net focussed interviews were completed with eligible children by one researcher (IN). These are qualitative interviews that incorporate both collaborative personal network mapping and conversation following a semi-structured interview guide. A template network diagram, or sociogram, is shown in Figure 1. Children were asked to write down (assisted if necessary) the people, things or places important to them, ranked in three tiers of importance. This is not an objectively validated data collection method for children, due to the paucity of social network analysis with this group. However the use of sociograms is thoroughly evidenced in child social work practice, and is simple low-technology approach, suitable for use with child populations (Hogan et al., 2007). Methodological developments indicate that using visual tools such as sociograms in qualitative or mixed methods social network analysis with children is best practice in research, emphasizing both structure and process as relevant aspects of social networks (Tubaro et al., 2016). As such the use of both network interview and visual mapping tools was determined to be the optimal means of data collection in this instance. Children were prompted to collaboratively create the network map using the phrase: “*I’d like to know about the people, places, activities and things that are important to you. The most important go in the middle, the ‘almost as’ important in the second circle, and the third most important go in the outside circle.”*

Interview questions investigated the quantity and quality of social ties identified, as well as the places, objects and activities valued by the child and the role of each in the child’s self-perceived wellbeing. Questions from the semi-structured interview guide after the initial prompts included follow up questions such as, *“What does this person do? How do they matter to you? Why did you put them in this circle?”.* Also included in the topic guide were name generator prompts for if children were struggling to spontaneously come up with ideas. These included, *“who would you talk to about something really important”* and *“if you had some really good news, who would you want to tell? Who else?”.* Children were prompted to include negative social ties through the prompt, *“Is there anyone who could be in the map, who might not be important in a good way?”.* Dynamic shifts were explored through questions such as *“What would you change? Would you change things?”* and *“Is there anyone/anything who used to be in this map, but isn’t any more? Why not? How is it different now compared to when they were around?”.* This semi-structured protocol allowed for exploratory questioning as new ideas arose, while generating information about quantity and quality of ties, important for generating thematic findings.

Interviews were completed at the site of the third sector organization aiding recruitment between August 2018 and March 2020. Audio recordings and network maps were retained by the research team as analysis material. Participants were unrestricted by type or number of network members. Children were encouraged to identify any people, places or things that were important to them, in a positive or negative way.

The team reached a consensus that data was likely saturated at 14 interviews, but 3 further were conducted to confirm that no further data was required as no significantly novel themes were generated from this point on. A sample of 12 or more interviews is considered sufficient for purposive, non-probabilistic samples (Guest et al., 2006). This is the typical sampling strategy used with hidden, stigmatized or hard to reach populations such as COPMI, including this study. Interviewers were not known to participants or recruitment organizations prior to data collection.

### 2.3 Data analysis

Inductive thematic analysis was applied to transcripts following Terry et al. (2017)’s guidelines. Interviews were transcribed verbatim by the interviewer (primary researcher). This facilitated the first stage of the thematic analysis process (familiarization). NVivo V.12 was used to code and search for themes in both transcripts and network data files. Words, phrases or sentences were coded with semantic/categorical labels (e.g., animals, emotional support, parental relationship). These were reviewed by the primary researcher with multiple readings to saturate coding as far as possible. This was determined when no new semantic labels were generated after multiple reviews.

Once coded, transcripts were distributed amongst the research team for checking, data immersion, and familiarization. Common clusters of code identified by individual team members and reviewed as a team. Themes were then generated through discussion and discrepancies were resolved through consensus in subsequent meetings. Interpretation of themes was cross checked between team members to promote reliability. The resultant thematic framework was applied to the remaining three transcripts conducted after this process. At this point themes were rereviewed and considered saturated as only minor alterations were required. These themes were concretely named, the final framework was appraised by the team and agreed upon as representative of the data set. At this point analysis was concluded.

### 2.4 Ethics

The University of Manchester (Ref: 2018-3572-6390) and Barnardo’s Research Ethics Committees approved the study.

## 3 Results

### 3.1 Sample characteristics

17 semi-structured network interviews incorporating personal network mapping were completed with children and young people at three sites in England spanning 1 year. Demographic information for this sample is provided in Table 1.

**TABLE 1:** Participant information.

Variable		n = 17
Gender	M	6
	F	11
	Other	0
Ethnicity	White British	16
	Other	1
Age	Mean (SD)	10.11 (3.84)
	Range	6–17
Site	1	11
	2	5
	3	1

A range of network members were identified by children. These included informal network members including parents, siblings, non-immediate family members and non-related adults and child peers. Formal connections included teachers and workers from third sector organizations. Non-human network members included animals, activity groups, solo activities, toys and characters. Parents were the most consistently identified network member, followed by animals.

### 3.2 Network features

A range of network members were identified by children. These included informal network members including parents, siblings, non-immediate family members and non-related adults and child peers. Formal connections included teachers and workers from third sector organizations. Non-human network members included animals, activity groups, solo activities, toys and characters. Parents were the most consistently identified network member, followed by animals.

Five core network features were interpreted from the data: (i) parents as primary providers of support (ii) limited networks and diminished connections over time (iii) substitutable ties including professionals (iv) peer connections as source of both support and strain (v) coping strategies of self-censorship, avoidance and animals. Each theme is presented descriptively and illustrated by direct quotations from the dataset. Participants are identified only by age and gender for anonymity reasons.

#### 3.2.1 Network feature 1: parents as primary providers of reciprocal support

Parents were the most commonly identified network member, with all children placing them as a primary tie even if the relationship was described negatively at points. Many children lived in single parent households, in every instance living with the ill parent. Varying degrees of involvement were attributed to the other parent. Identified parents, and all parents living in the same home as the child, were described as the primary providers of reciprocal support. Some parents living outside of the child’s primary home were also described as a primary, supportive network tie.

*“[when completing the ego-net map] I’m doing mum first because she’s the most importantest to me right now. Because she’s the one that protects me.”*

- 6 year old girl

Children received the most assistance from their parents, but also described assisting their parents in various ways. Most children described their parent or parents as providing both instrumental support (i.e., tangible assistance) and emotional support to them.

*“Mummy and doggy” [in response to initial prompt asking who their most important ties are]*

- 7 year old boy

Relationships with parents were described less positively by older participants and by children who provided a substantial caring role to the parent. These two factors were congruent, as older children typically have increased capacity to support parents, particularly instrumentally.

*“My mum can’t eat much food so I have to make things certain ways, and I*

*constantly cook for the house… I like seeing my mum enjoy food because she can’t eat it a lot of the time”*

- 17 year old girl

The caring role was described as a source of strain by many children in the study. This was the case even when the parent was receiving professional care to meet their support needs including mental health treatment.

*“Stressful. Trying to look after my mum and trying to get all my work in on deadlines. Losing coursework because of my mum’s carers… I was doing coursework for 4 h and one of the carers refused to wash my mum and take her to the toilet.”*

- 17 year old girl

Children demonstrated limited understanding of their parents’ illness, and described exclusion from involvement in their parents’ care plan and treatment, despite contributing significantly to their care. Younger children were less likely to express a desire for further information on their parents’ illness whereas older children described frustration in being invisible to involved adults including professionals. Nonetheless, most children still described their parents as one of their most important ties and an invaluable source of emotional support in the context of the difficulties they were facing, even though their parent’s mental health issues were acknowledged as potentially disruptive to their capacity to provide emotional support.

*“My mum because no matter what, she’s really understanding all the time… I don’t know. I think it’s because my life isn’t that pleasant. I don’t really have a normal family. I have a weird family. And my mum suffers from mental health. I suffer from extreme anger issues.”*

- 16 year old girl

*“I tend to talk to my dad or mummy, my mum, even if she is not in a good mood”*

- 13 year old girl

#### 3.2.2 Network feature 2: limited networks and diminished connections over time

Younger children of primary school age (11 and below in England) often described a range of supportive ties besides parents. These included extended family or non-related adults such as neighbors or family friends. These children did not describe sufficient peer connections however, even if adults were present in their network. This was considered a limitation of their network by many children of all ages.

*“Yep, more friends… I’ve only got six [on what he would change].”*

- 9 year old boy

*“I did have other friends that lived in the street but they’ve moved away and I don’t speak to them that much anymore.”*

- 17 year old girl

Older children and teenagers were less likely to describe the same breadth of support from family and other adults, and many expressed dissatisfaction with the level of support they received. This was particularly the case if they had access to more support previously. Multiple children described the loss of informal network connections over time, of both adults and peers, both family and non-family. In particular children described a loss of instrumental support over time. Ties became less involved as children required less instrumental support.

*“My brother still doesn’t come over and help. He’s just sort of kept himself to the side. I suppose when I was younger he was there, like he picked me up from school and stuff.”*

- 17 year old girl

Many reasons were provided for loss of ties over time. These included bereavement as older adults died, moving home or school, as well as changes in family or family friend dynamics. Dynamic shifts were attributed to conflict and disagreement including dissolution of parent’s romantic relationships, or gradual loss of contact. Some young people described previously highly valued family ties diminishing in closeness over time.

*“I can see them [extended family] anywhere, but they don’t bother with me. Like my dad’s side don’t bother with me as much.”*

- 13 year old girl

Younger children described primarily family ties as most highly valued. Teens often had an equal focus on peer ties. The shift from adult connections to peer connections was associated with a loss of instrumental support previously provided by adult ties in many cases.

#### 3.2.3 Network feature 3: substitutable ties including formal ties to professionals

A typical feature of vulnerable children’s networks is that alternate ties can provide compensatory support if primary ties are limited in availability or capacity. Many parents with SMI experience at least episodic limitations in parenting capacity. This, combined with the limited and/or diminished networks identified indicate that COPMI are likely to benefit from compensatory support where available.

Children who were identified as COPMI by formal network members such as teachers and educational professionals reported receiving highly valued instrumental support from these sources. Flexibility and understanding regarding the competing commitments of caring role and academic commitments were valued. These teachers were also described as providing a degree of emotional support.

*“She [teacher] knows when I’m out and just knows my ability to work and knows situations at home and how I get when I’m distressed and tearful and stuff… recently her and [other teacher] have helped me a lot based on my own mental health. Because I have anxiety. It’s not that bad but it can get bad sometimes, if I’ve had a lot- I had a meeting with them yesterday at lunchtime actually, and we talked about how they can help, help with things at home and school.”*

- 15 year old girl

Services that could interact with the school in an interdisciplinary fashion were also described as valuable. Case workers and advocates from third sector settings, and social workers and counselors in school were described as providing support to children.

*“They have meetings [caseworkers and advocates]… and just talk about how we are, how they can help us… they like fight my corner when I’m not there… and they were like, no, this girl has this situation that she’s in… I’m sure if she didn’t have the situation she’d be earlier every single day, but this is the reason. So that helps a lot.”*

- 13 year old girl

However, many children reported that they had not been identified as in need of additional support due to COPMI status in school. These children reported significant difficulties with the lack of flexibility and understanding afforded to them due to the conflicting pressures of their caring role and of academic pressures.

*“So when I had anxiety before and I was off [from school] with my mum because she was ill and that, they weren’t very accepting, they were just ringing me up and shouting at me for not being in.”*

- 17 year old girl

*“So the teachers saying ‘she shouldn’t be doing this’ and ‘she shouldn’t be doing that’ and ‘she shouldn’t come in late’, when that first happened in year nine coming in late she [the teacher] said I should get detentions for it.”*

- 13 year old girl

For those children who were eventually identified as COPMI by the school, formal ties were able to start to compensate for some of the difficulties experienced. Participants reported improved wellbeing in school, including a reduction in stress due to competing roles, once this support was in place.

These findings indicate that formal ties can provide compensatory support where informal support is diminished, but that this is dependent on quality of identification and responsiveness. At times, children described professionals including teachers as facilitating emotional and recreational support such as groups or activities designed to promote friendship between children.

*“It’s just called friendship club, but to make it easier you can just call it [teacher]’s club. She is my teacher.”*

- 8 year old girl

*“Because people know about the things some of it goes a bit wrong because some people tell other people. But most of it has gone quite well because she [school support worker] does it so like, she knows who will tell people and who won’t tell people. So she gets the people who won’t tell people first and gets a big group of them. And then people she thinks might will tell somebody she just does on their own or in a partner so they don’t tell anyone else. So I’ve got her to talk to as well.”*

- 10 year old girl

Children and teens described receiving valued emotional support through peer ties, as is typical as children develop extra-familial ties over time. Peer emotional support was deemed particularly valuable when family support was limited. However these were insufficient to meet all their needs and fully compensate for a lack of adult support. The supportive role of peer connections is described further in section “3.3.4 Network feature 4: peer connections as source of both support and strain.”

#### 3.2.4 Network feature 4: peer connections as source of both support and strain

Peer relationships were described as a central source of closeness and support for older children in particular. However, peers were described as sources of both support and strain at times for COPMI. Where close friends were available, they provided a significant source of emotional and recreational support for children of all ages, alongside both group and solo valued activities.

*“Because he’s my bestest, bestest, bestest bud. And what makes him such a best bud. He’s really nice and he sticks up for everyone.”*

- 9 year old boy

*“Because I can always talk to [friend] and she makes me happy when I’m always sad sometimes”*

- 6 year old boy

However, friendships could become problematic related to trusting and secret keeping. Breaches in confidentiality, typically due to parental mental illness and the related stigma.

*“Yeah because I don’t really trust them [former friends] anymore because everybody tells everyone everything. And it’s just a bit like… mm, not what you want.”*

- 10 year old girl

This stigma was a source of challenge for children in many of their peer relationships. Some participants anticipated significant difficulties in this aspect of trust in friendships due to prior experiences.

*“No. I just don’t want, you know, if I do get in and argument with them [friends], I don’t want them to go talking to anyone else about it. And I don’t want that being spread around. Because, let’s be honest, when you do get in an argument, and you have chose the wrong friends, they will start telling people about certain things.”*

- 15 year old girl

The strategies used to navigate trust difficulties are described in section “3.3.5 Network feature 5: coping strategies of (i) self-censorship (ii) avoidance and (iii) animals.” Furthermore, some children described difficulties devoting time to friendships and recreation due to their caring roles.

*“That’s my time filled up, I don’t even go out”*

- 16 year old girl

*“I like get her prescriptions, sort her medicines out, sort that out, sort out her appointments with the doctor and nurses, clean up for her, tidy up for her, do tea, I have to ring up with her and stuff like that”*

- 13 year old girl

Peer connections were complex, at times limited in accessibility, and required sophisticated navigation strategies described below. Digital platforms and messaging facilitated remote contact and helped sustain valued connections impacted by caring role.

#### 3.2.5 Network feature 5: coping strategies of (i) self-censorship (ii) avoidance and (iii) animals

The themes presented so far depict some of the difficulties faced by COPMI, which impact their need for, and access to support related to parental SMI. Children described a range of strategies used in their network interaction to mitigate some of these difficulties. These can be termed ‘network navigation strategies’. Self-censorship, the avoidance of potentially stigmatizing topics such as parental SMI was commonly utilized by children. This typically was a learned behavior developed in response to negative past experiences with sharing information about their family with peers, as described in the prior theme. Participants described learning to be selective in their disclosures with both peers and adults and both informal and formal connections.

*“I just like keeping things to myself and never talk to anyone…”*

- 7 year old boy

This included disclosures within and outside of the family where negative repercussions were anticipated.

*“I just don’t like telling them [family] anything. Like if I have a fight in school, mum finds out because she talks to the teacher.”*

- 7 year old boy

Significant levels of trust were required before children would divulge sensitive information. A school-based group intervention with a trusted adult facilitator who helped guide children through this dynamic was particularly valued. Valued friendships those with high levels of trust with regards to confidentiality of disclosures. These peer ties were described as significant sources of emotional support. When there were no such friendships identified, emotional support was usually limited. Some children avoided friendships entirely because of trust difficulties, others described intentionally ending friendships due to confidentiality breaches, and one avoided friendships almost exclusively, identifying this as positive for his well being.

*“So I used to talk to her [friend] quite a bit but then she told everybody everything I told her, so then it was a bit like, ‘well bye then’.”*

- 10 year old girl

*“He’s my only friend really. Good. Because then I don’t have lots of people to bother me.”*

- 7 year old boy

Almost every participant described one or more animals as primary ties. Dogs in particular were described as notable sources of companionship.

*“Whenever I’m upset my dog’s always there so I can just stroke my dog and stuff.”*

- 11 year old boy

Animals were described as providing emotional support, valued particularly where emotional support from other network members was limited.

*“Say I’m doing stuff with my mum, I’ll just go sit with him. And if I’m really sad he’ll just like calm me down.”*

- 13 year old girl

*“It’s just nice having him there if I feel down or whatever. Sometimes I can’t talk to my mum because maybe I’ve had an argument with my mum but I’ve got the dog.”*

- 17 year old girl

Limitation in lifespan or loss of pets due to unstable housing was a source of sadness for some. Pets were also described as a source of support for the ill parent, which reduced the child’s perceived caring burden described in 3.3.1.

## 4 Discussion

The social networks of children of parents with SMI are characteristic of the networks of vulnerable children in several ways, with some key divergencies (Nevard et al., 2021). COPMI describe networks that are at points impoverished in terms of the limitation of ties, and the diminishing of connections over time. As typical of vulnerable child populations, parents are primary ties and sources of instrumental and emotional support. However, alternate ties can provide substitute benefit providing networks are suitably diverse. Distinctively, this child group provides corresponding instrumental and emotional support to parents in an inversion of typical filial relationships, increasing with age, which could engender additional support needs over time. Additionally, impoverished networks, particularly if diminishing longitudinally, indicate that appropriate substitute or additional support are not always available to the child.

### 4.1 Theoretical implications

From a theoretical standpoint, this study conceptualizes networks in terms of personal communities with a specific focus on support flows. The mechanistic effect by which these network structures allow for resource flows are not clearly elucidated and are more broadly a subject of theoretical contention in health research. Two, potentially complementary, approaches to explain the positive effect of network embeddedness are the main-effect model and stress-buffering model (Kawachi and Berkman, 2001). The former posits that social relationships inherently have positive effects, whereas the latter states that social relationships mitigate negative effects for children under stress. Both are conceptually coherent for COPMI as a population. Furthermore social networks can mechanistically work to have negative impacts, in terms of contagion of negative behaviors or ‘cross-level interactions’. These are where individual characteristics negate the positive impacts of overall social capital. This study found the impact of stigma to be a negative component of network integration at times.

Commonly explored phenomena in social network analysis in mental health research are health related behavior contagion, resource flow and health information flow as well as intergenerational transmission of SMI. Proposed causal mechanisms for health and wellbeing changes within networks can include level of homophily between members, access to instrumental, emotional, informational support, access to recreation or the contagion of behaviors, emotions and illnesses (Thoits, 2011; Christakis and Fowler, 2013; Montgomery et al., 2020). This study does not identify which causal mechanisms are specifically in operation for COPMI, but access to support and valued companionship (or lack thereof) were the key ways in which children described networks impacting their quality of life.

Children describe actively generating coping strategies in order to manage the sources of strain within their networks. The findings of this study support a conceptual position that children are active network navigators rather than passive subjects of network effects. As such, mechanisms which situate actors, including children, as active co-constructors of networks are supported by this study. This corroborates other critiques of conceptual frameworks surrounding this population. Research that relies too heavily on the binary of risk versus resilience can frame children as purely passive actors, subject to the external factors that facilitate increased risk and promote resilience. Furthermore, these types of research can construe children as half-formed subjects (i.e., adults in the making) rather than as discrete whole individuals with a range of competencies and abilities to navigate their own life situation with a degree of autonomy (Gladstone et al., 2006). This research centres children’s voices on an ethical basis and construes children as experts on their own experiences. However, it also evidences children as active expert managers of these experiences.

### 4.2 Practical implications

The strategies employed by COPMI were not always effective and could prove detrimental long-term. Previous systematic reviews demonstrate that network embeddedness has positive wellbeing outcomes for vulnerable children. Participants in this study described intentionally limiting peer networks as a coping strategy which could prevent them from accessing beneficial network ties (Nevard et al., 2021). If children are not supported in creating positive relationships and developing adaptive network navigation skills, these coping strategies risk entrenchment. Long term issues in network integration could have effects well into adulthood as access to social networks is overall protective, despite the fact that these strategies were initially developed in order to address negative network effects (Hare Duke, 2017). Potential applications that assist children in developing network navigation skills are provided in section “4.3 Application and further research.”

Children describe a number of coping strategies employed to manage the strain involved in having a parent with SMI. Some directly addressed caring burden but more frequently strategies related to social stigma or a lack of perceived support for the family unit or the unique challenges they faced. Recreational support including peer connections and valued activities or hobbies were considered important. However, difficulties with trust, linked to the particular social stigma this group is exposed to, may prevent children from utilizing peer relationships to the extent that other vulnerable child groups do.

These difficulties are actively managed by the child, either by selectivity sharing information, or avoiding relationships entirely, a strategy likely to maintain and reinforce the impoverishment and shrinkage of networks. Animals play a key role in providing emotional support to children, and sometimes parents, although the possible contribution to the young person’s caring load should also be noted. A plausible inference as to why animals were so central in the networks of this population, a phenomenon not previously noted for vulnerable children more broadly, is the lack of trust and stigma navigation required in these ties. Valued animals were reliable sources of emotional support, although not always reliable in their longevity as connections due to lifespan, which could exacerbate network shrinkage. The companionship role of pets reflects previous findings in vulnerable adult populations (Brooks et al., 2016, 2018).

### 4.3 Limitations

Data was drawn from a moderate sample of 17 participants. This was considered suitable for preliminary exploratory research due to perceived saturation of data at 14 interviews, and a projected saturation at 12 interviews (Guest et al., 2006). A lack of ethnic diversity and uneven gender distribution in the sample also represents a difficulty in extrapolating findings across populations even within England. This was identified by the research team at the time of data collection, however the dependence on intermediary recruiters limited influence over sample demographics. Focused research on these demographics could be a valuable direction for future work. The purposive sampling strategy utilized also meant that all children were already known to third sector organizations and so were unlikely to include the most vulnerable subsections of the demographic. This included COPMI who have not been identified as such outside of the family, which is overall an issue in the field for both research and intervention. However, researchers intentionally recruited from a range of organizations, beyond only young carer groups, and consequently represents a more diverse group than some comparable research which only conceptualizes COPMI within their caring role.

Self-report of psychiatric diagnosis by parents prevented researchers from assessing and confirming adherence to inclusion criteria directly. However, this was a necessary adaptation of the recruitment strategy in response to reports by families and intermediaries of a lack of access to necessary mental health service provision for SMI. This lack of service provision is in itself likely to have an impact upon COPMI, parents and their wider networks, impacting the findings of this study.

No children under six were included in this study; they are underrepresented in qualitative research overall, a noteworthy exclusion in research that aims to centre the voice of hidden or under-identified populations on an ethical basis. However, the methodological limitations of social network interviewing make it challenging to collect interview data from younger children. While parental report could be used as a proxy means of data collection for these children, we chose to prioritize children’s voices in the research by directly collecting data from children we were able to interview effectively.

The validity of findings could have been further evidenced through a process of respondent validation, or further interviews with additional children to elicit feedback on preliminary themes identified. However, opportunities for respondent validation were limited due to closure of recruitment organizations during the COVID-19 pandemic.

### 4.4 Application and further research

A recent systematic review has identified a striking lack of qualitative data on networks of children of parents with SMI (Nevard et al., 2021). This is the first study to our knowledge to present descriptive network data regarding the configuration, dynamism and function of social networks for this population. This study demonstrates that qualitative egocentric social network analysis is an appropriate and effective way of generating network findings with vulnerable child groups. Best practice in type and timing of interventions for vulnerable children including COPMI is contested (Bee et al., 2014; Bee, 2015; Reupert and Maybery, 2015). This study provides preliminary conclusions regarding optimal intervention features on an empirical basis.

Young carers often report need for respite as a priority in available interventions. This research is in keeping with our finding that recreational support with peers is an important resource that COPMI receive through social networks. Groups or holiday breaks specifically designated for young carers have the advantage of improved trust amongst similar peers, a valued resource reported by participants in our study who often reported poor trust experiences with non-carer peers. However, a diversity of ties is also likely to be key, as children report valuing a range of diverse support flows; homophily of networks can also present problems. As such, it is not sufficient to provide respite to facilitate artificially created peer networks; interventions must also focus on strengthening and enhancing pre-existing organic networks. A further consideration with respite and recreation is whether it has a substantive, ongoing impact on children’s networks. If interventions are provided as a one off, they may temporarily provide recreational support, but have no long-term positive impact on the peer networks of the service users.

Integrated family interventions that equip children with network navigation skills should be provided as early intervention before maladaptive strategies are embedded or network shrinkage takes effect. This relational skills coaching could focus on identifying useful social ties, promoting help-seeking behaviors, managing stigma and its impact, fostering healthy communication and maintaining relationships in challenging circumstances. These approaches could help foster network support flows and offset the impact of negative experiences before they lead to maladaptive beliefs or antisocial behavioral patterns. Relevant psychoeducation will also be necessary as the child develops cognitive capacity to engage with conceptual ideas including SMI, stigmatized relationships and different social roles. These could be developed in tandem with parenting interventions, a similarly under resourced intervention area (Dunn et al., 2023).

Effective network level interventions for this group would also integrate ongoing access to effective formal substitute ties to offset lack of support from informal networks, including from parents. This is likely to be particularly necessary for older children due to network shrinkage over time. Preempting transitions that are likely to impact networks, such as school changes or changes in accommodation, may be more protective than reactive responses. Interventions could also facilitate access to peer ties COPMI may be more likely to consider trustworthy (e.g., with other young carers or vulnerable children).

Participants consistently reported the importance of animals in personal networks. These animals were usually described as providing an emotional support function to the child, or less often to a parent. This latter would have a proxy effect on the child, reducing their own care burden. Some children even reported pets of family members or neighbors as serving an emotional support function, indicating that an animal need not belong to the family to provide emotional support to the child. Animals provide a number of advantages as a social connection. These include reliability in companionship, a lack of stigma or judgment and touch based bonding including comfort, relaxation and reciprocity. Health research demonstrates that pets promote health and counter isolation. This indicates a potential role for therapy animals, or emotional support animals, in network interventions for vulnerable children including COPMI. Downsides include restriction to emotional support functions, and instability of pet ownership for children who have lack of agency over factors such as parental divorce and changes to accommodation, as well as the limited life spans of most animals. Pet ownership also introduces further care burden to the child. Therefore, while we can consider pets to present a good avenue for emotional support substitution, this is only one component of a human social connection that cannot be fully replicated or substituted for. Animal based interventions would not be sufficient network interventions in and of themselves.

## 5 Conclusion

This study demonstrates the need for improved and integrated interventions for this COPMI, with an explicit focus on skills-building and network navigation, in addition to the more traditional psychoeducation and respite. Consultation with the various mental health, social care and educational professionals that work with this population will be instrumental in the development and formulation of potential interventions for this demographic. The role of animals in network interventions merits further investigation, specifically regarding the balance between emotional support function and increased care burden.

## Data availability statement

The datasets presented in this article are not readily available because raw interview data is not provided on the basis of confidentiality of minor participants. Requests to access the datasets should be directed to IM, imogen.nevard@manchester.ac.uk.

## Ethics statement

The studies involving humans were approved by the University of Manchester Research Ethics Committee and Barnardo’s Research Ethics Committee. The studies were conducted in accordance with the local legislation and institutional requirements. Written informed consent for participation in this study was provided by the participants’ legal guardians/next of kin for minors under 16 years. Written informed consent was obtained from the minor(s)’ legal guardian/next of kin for the publication of any potentially identifiable images or data included in this article. Written informed consent for participation in this study was provided by participants 16 years and over. Written informed consent was obtained directly from participants 16 and over for the publication of any potentially identifiable images or data included in this article.

## Author contributions

IN: Writing – original draft, Writing – review and editing, Conceptualization, Data curation, Formal analysis, Investigation, Methodology, Project administration. JG: Writing – original draft, Writing – review and editing, Supervision. HB: Writing – original draft, Writing – review and editing, Supervision, Methodology. PB: Writing – original draft, Writing – review and editing, Supervision.
